# The physical environment matters: room effects on online purchase decisions

**DOI:** 10.3389/fpsyg.2024.1354419

**Published:** 2024-06-18

**Authors:** Ann Eklund, Anna Edenbrandt, Johan Rahm, Maria Johansson

**Affiliations:** ^1^Environmental Psychology, Department of Architecture and Built Environment, Lund University, Lund, Sweden; ^2^Department of Economics, Swedish University of Agricultural Sciences, Uppsala, Sweden

**Keywords:** goal framing theory, priming, sustainable consumption, choice experiment, online behavior

## Abstract

**Introduction:**

People as individual consumers are regularly targeted in sustainability campaigns or communications with the hope of enhancing sustainable behavior at an individual level, with subsequent sustainability transformation at a larger societal scale. However, psychological motivation is complex and campaigns need to be based on an understanding for what individual, and contextual, factors support or hinder sustainable behavioral choices.

**Methods:**

In a discrete choice experiment, participants made hypothetical online purchases in each of three rooms designed to evoke associations to *hedonic*, *gain*, and *normative* goal frames. Participants were shown a campaign message intended to prime sustainable textile consumption prior to the purchase. For each product (t-shirt or bananas) hedonic (comfort/look), gain (price), and normative (organic/ fairtrade) attributes were varied in an online choice experiment.

**Results:**

Preferences for the normative attribute of t-shirts increased in the normative room compared to the room with gain associations. No effect of the rooms with hedonic or gain priming was observed on the choice.

**Discussion:**

The study supports the hypothesis that the physical room can enhance goal frame activation and behavioral choice but concludes that such priming effect is sensitive to specificity of the prime.

## 1 Introduction

The world is facing unprecedented environmental challenges with biodiversity loss, chemical pollution, and climate change following human population growth and overconsumption of resources (IPBES, 2019; IPCC, 2023). To halt environmental degradation, an urgent transformation to sustainable cultures and societies is needed (Oskamp, 2000; Winter, 2000; Clayton et al., 2016). While political decisions and policies play an essential role in the transition towards a sustainable society, the individual motivation and behavioral change is critical for collective action, not least when it comes to the consumption of goods (Amel et al., 2017). People as individual consumers are regularly targeted in sustainability campaigns or communications with the hope of enhancing sustainable behavior at an individual level, with subsequent sustainability transformation at a larger societal scale (McKenzie-Mohr, 2000; Fischer et al., 2021). What these campaigns essentially rely on, is that people are guided by their internal motivation to make sustainable behavioral choices, and that this motivation and behavioral change can be influenced by soft policy measures such as campaign messages (Schuitema and Bergstad Jakobsson, 2013), preferably combined with other behavioral intervention strategies (Rau et al., 2022). However, a first step towards success is to acknowledge that psychological motivation is complex and for campaign messages to induce behavior change, a good match between people’s motivation, the campaign content, contextual factors, and the desired behavior to be obtained is needed (Geller, 2002). Therefore, campaigns need to be based on an understanding for how the campaign content is perceived and appraised by the consumer and what individual, and contextual, factors support or hinder sustainable behavioral choices (Oskamp, 2000; Janiszewski and Wryer, 2014; Steg et al., 2016).

People are constantly processing their surroundings in relation to prior knowledge and their value orientation. Thus, when an individual interacts with a sustainability campaign message, the message will be processed by the individual, and a subsequent behavioral action will depend on how the message is understood. In their 2007 paper, Lindenberg and Steg introduced an overarching framework of psychological processing based on previously established models, the Goal Framing Theory (GFT). The framework describes three overarching *goal frames* to guide human behavioral choices in relation to sustainability, through which any given situation is processed in relation to a multitude of personal goals. These three goal frames are the *hedonic* goal frame in which the person seeks pleasure (or to avoid effort) and feeling better instantly, the *gain* goal frame in which the person seeks to maximize the personal resources (such as money and status) and the *normative* goal frame in which the person seeks to “act appropriately” (Lindenberg and Steg, 2007).

Each of the three goal frames may be activated within the individual at any given time, but one goal frame will be focal and guide behavior in each situation, for instance when making a purchase (Steg et al., 2016). In relation to environmentally friendly behaviors, Lindenberg and Steg (2007) identify the normative goal frame as the most likely focal goal frame. More recently, empirical studies have supported the notion of a link between a focal normative goal frame and sustainable behavior (e.g., Gerhardsson et al., 2019; Thøgersen and Alfinito, 2020; Bolos et al., 2022). Considering sustainability campaigns, they likely seek to activate the normative goal frame as associated with concern for the environment and other people.

The accessibility of each goal frame is to some extent tied to the individual’s values (do Canto et al., 2023); strong self-enhancement values are associated with hedonic and gain goal frames, and strong self-transcendence (altruistic and biospheric) values are associated with a normative goal frame (Stern, 2000; Steg et al., 2016). Values are trans-situational, appear relatively stable, and are expected to influence the accessibility of a certain goal frame across situations (Schwartz, 1994). Nevertheless, situational contexts may cause a goal frame that is not directly aligned with the individual’s value orientation to be focal temporarily (Steg et al., 2014; do Canto et al., 2023). For instance, when an individual is interacting with a sustainability campaign, the normative goal frame may be temporarily activated. In parallel, the person may also interact with other stimuli in the physical and social environment that he or she resides in, which could influence which goal frame becomes focal. However, research is needed to shed light on how external factors and situational conditions may impact the activation of a focal goal frame as a more temporary state (Steg et al., 2016; do Canto et al., 2023).

Prior research has shown that a focal goal frame may be primed by stimuli in a situational context. Thøgersen and Alfinito (2020) used text primes and found these to influence choice of tomatoes in an experimental setting. From an environmental psychology perspective, it may also be reasonable to assume that similar priming effects on focal goal frames could be induced by the physical environment in which a person resides when making a purchase, since people continuously perceive and appraise their surrounding physical environment (Küller, 1991; Gärling, 2014). Early and contemporary environmental psychology research has revealed that the perception of interior design and aesthetics of a room influence how objects, or other persons, are appraised (Maslow and Mintz, 1956; Miwa and Hanyu, 2006) and how communication is undertaken (Gifford, 1988; Miwa and Hanyu, 2006). The room environment is appraised by the individual for its colors, light, and materials, and environments can be perceived differently on various environmental dimensions (Küller, 1975; Küller, 1979; Bell et al., 2001). It has also been shown that while normative information alone may not be able to activate a normative goal frame with subsequent sustainable behavior, the behavior may also be influenced by cues in the environment (Keizer et al., 2011).

Existent literature also provides examples of how the physical environment can prime specific behavior. The sight of a library environment causes people to lower their voices (Aarts and Dijksterhuis, 2003), and business-related items induce more competitive behaviors (Kay et al., 2004). In relation to consumption, contextual primes (e.g., dollar signs versus clouds in the background of an online shop) lead people to prioritize price versus comfort in simulated online purchases of cars and sofas (Mandel and Johnson, 2002), behaviors linked to gain and hedonic goal frames (Lindenberg and Steg, 2007). Multiple studies on consumer behavior have also evaluated the influence of ambient environmental factors such as light, music, scent, color, cleanliness (e.g., Turley and Milliman, 2000; Mari and Poggesi, 2013) on various other outcome variables including purchase intentions, pleasure and willingness to buy, or willingness to pay in physical shops (Baker et al., 1992; Brengman et al., 2012; Doucé et al., 2013; Doucé and Janssens, 2013; Doucé et al., 2014; Quartier et al., 2014; Sunaga et al., 2016; Guido et al., 2017; Elmashhara and Soares, 2020; Hwang et al., 2020; Kim and Kim, 2020).

More recent developments in consumer research include studies of contextual congruity (e.g., Mattila and Wirtz, 2001; Doucé et al., 2014; Sunaga et al., 2016), i.e., the interaction between two or multiple physical environmental cues’ effects on behavior (Mari and Poggesi, 2013). Another line of research is the investigation of the environment within virtual servicescapes (Mari and Poggesi, 2013; Neves et al., 2024), including comparisons to physical stores (e.g., Pizzi et al., 2019; Lombart et al., 2020). As online shopping is experiencing an increasing trend (Eurostat, 2022a) it is of interest to research the psychological processes at the interface between the physical and the online environment that the person is interacting with. Instead of focusing on either online or physical environments separately, considering the possibility of priming a focal goal frame for an online consumer (Mandel and Johnson, 2002; Thøgersen and Alfinito, 2020), depending on the contextual congruity (or incongruity) of the physical environment that the person resides in when making purchases online. These environments could represent two or more competing, or congruent, environmental (physical or digital) primes that the person interacts with simultaneously (Bargh, 2006). With an increasing urgency for transformation towards sustainable societies, sustainability campaign messages intend to prime a normative goal frame, while the impact of environmental primes may cause the normative focal goal frame to shift to a gain or hedonic focal goal frame (Lindenberg and Steg, 2007). As the focal goal frame is expected to guide the behavioral choice of the consumer, the success of a campaign may hence depend on the congruence with the environment that the person is in.

**Figure 1:**
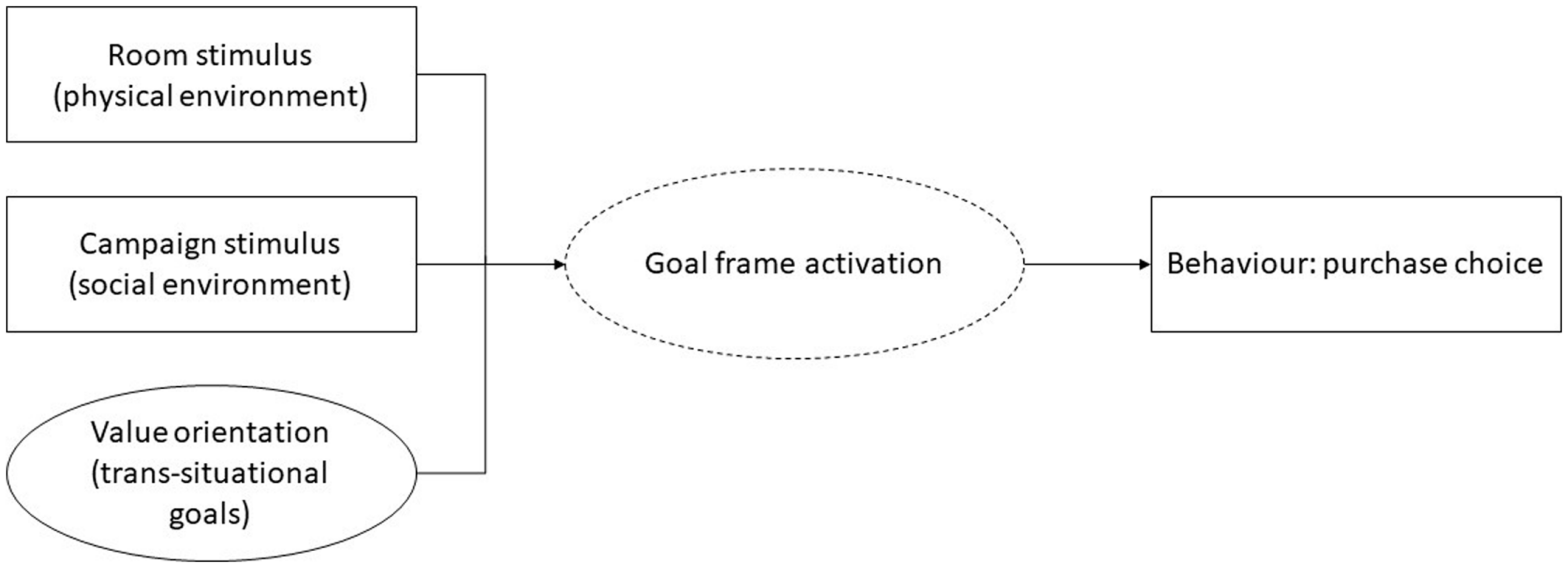
Model for the priming of goal frame activation generated by an interaction between the campaign stimulus and room environments, guiding purchase choice. Pre-existing values are believed to influence the salience of goal frames and is controlled for. Dotted lines indicate that goal frame activation is not measured *per se* but is implied by the purchase choice.

To further an understanding for how congruent or competing stimuli in the physical and online environment may influence goal frame activation and online purchase choices, this study addresses the question: *Can the room environment affect online purchase decisions among participants who are also targeted by an online campaign stimulus for a sustainable choice?* To answer this question, we designed a quasi-experimental study where participants (1) interact with an online sustainability campaign, intended to prime a normative goal frame, prior to (2) performing a simulated online purchase choice of products with hedonic, gain, and normative attributes, and (3) while being seated in three different physical room environments intended to prime hedonic, gain, or normative goal frames, respectively. The model is illustrated in Figure 1. It should be emphasized that the intention is not to test the impact of the campaign *per se*, rather the campaign is introduced and kept constant to participants to ensure that all participants experience the same campaign prior to the experiment.

We hypothesize that the relative preference towards hedonic, gain, or normative attributes of a product will be greater in the room with the corresponding goal frame priming than in rooms with priming for other goal frames.

Moreover, in a complementary analysis we control for the participants’ orientation towards self-enhancement values, respectively, self-transcendence values.

## 2 Materials and methods

### 2.1 Development of the experimental study

To test the hypotheses of the study, three controlled “home-like” rooms were created in full-scale in the Lund University full-scale lab, at the department of Architecture and Built Environment. In each room, differently designed with associations to the three (hedonic, gain, and normative) goal frames, participants were shown a sustainability campaign stimulus, before making purchase choices in a digital discrete choice experiment (DCE) on an iPad.

The experimental design was achieved through interdisciplinary collaboration between researchers from the fields of environmental psychology (for the potential room effects) and economy (for the choice experiment design). Development of the experiment was undertaken in several steps, including scoping focus group interviews with campaign makers to ensure the relevance of the study and identifying the target audience for their campaigns. Furthermore, various aspects of the environments and the choice experiment (summarized in Table 1) were piloted prior to the data collection of the main study. The first pre-test of room interior design was tested using digital mood-boards and systematic investigation of the visual impressions by the Semantic Environmental Description (SED) scale (Küller, 1991). Rooms were then built in full scale and partly decorated according to the same style as the mood-boards. To validate the ecological validity of the rooms in the laboratory setting, as well the potential of the rooms to act as distinguished primes for goal activation, a convenience sample of 19 people visited the rooms when the rooms were partly decorated. Furthermore, pictures of the rooms were used in a digital survey where an additional 19 persons perceived the interior design through images. Both samples responded to the SED (Küller, 1975, 1979), Swedish Core Affect Scale (SCAS; Västfjäll and Gärling, 2007) and the Perceived Restorativeness Scale (PRS; Hartig et al., 1997). The pre-tests indicated that the rooms were not perceived as extreme environments, but that the rooms evoked statistically significant differences in associations to hedonic, gain, and normative goal frames (see Appendix A for detail).

**Table 1:** The different steps of developing the study.

Step	Test	Measures used	Sample
*1*	**Focus group meeting** *Digital focus group meeting with campaign makers*	Qualitative inquiry regarding relevance of the study, target audience of campaigns, and main aims of campaigns.	*n* = 6 (campaign makers)
*2*	**Mood board** *One-frame digital collage of room interior items*	SED	*n* = 6 (colleagues)
*3*	**Room interior** *Partly decorated rooms in the full-scale lab*	PRS SED SCA Goal Framing Associations	*n* = 19 (convenience sample of department staff, external collaborators, students, family members)
*4*	**Room interior digital** *Digital photograph of partly decorated rooms*	PRS SED SCA Goal Framing Associations	*n* = 19 (participants of the Mistra research programme consortium, and students)
*5*	**Choice Experiment** *Digital pretest of products as a choice experiment: t-shirts and socks*	Discrete choice data Follow-up questions on perceptions regarding choice tasks	*n* = 37 (convenience sample of colleagues, friends and family)

In the development of the DCE a pilot study was undertaken in which respondents selected t-shirts and socks in an online experiment. Following the choice experiment respondents provided information about how they perceived the choice tasks. Based on this information we made a number of adjustments to the experiment included in the main data collection. For instance, we included a lower price t-shirt without branding (ecological, fairtrade, and soft material) as pilot study participants highlighted that this would be the most realistically available product within the category. Another key conclusion from the follow-up questions in the pilot was that the normative labels (organic and fair trade) are not characteristics that consumers are used to see or reflect upon in their purchases of textiles. In the main data collection, we added a choice experiment with bananas, where consumers are more familiar with the normative attributes (organic and fair trade), and which is a product that many consumers purchase often. Similar studies on priming have used food items such as tomatoes (e.g., Thøgersen and Alfinito, 2020; Bolos et al., 2022), and the inclusion of bananas as purchase objects connects to this prior research.

None of the participants in any of the pre-tests participated in the main study.

### 2.2 Participants of the main study

A total of 88 persons participated in the study, 50 identified as female and 38 as male. The average age was 22 years (range 18–33). Most participants were students (*n* = 82), while some were either working (*n* = 4) on sick leave (*n* = 1), or currently unemployed (*n* = 1). Focus group meetings with campaign makers in preparation of the study (Table 1), informed the recruitment of young adults to match the age of the sustainability campaign’s target audience. Recruitment was mainly undertaken at the Lund University campus and to a smaller extent through convenience recruitment. Although the recruitment did not specifically target a student sample, a large proportion of persons within the target age in the university town were students. The sample size of 88 was based on the minimum number of participants needed for an effect size of d = 0.39 (Lindeman and Verkasalo, 2005), as calculated using the G*Power software (Faul et al., 2007). Furthermore, a power analysis in Ngene, based on estimates from the pilot study choice experiment, revealed less than 10 respondents were needed for main effects. The study and the sample size were approved by the Swedish Ethical Review Authority prior to recruitment and data collection.

### 2.3 Materials

#### 2.3.1 The room stimuli

**Figure 2:**
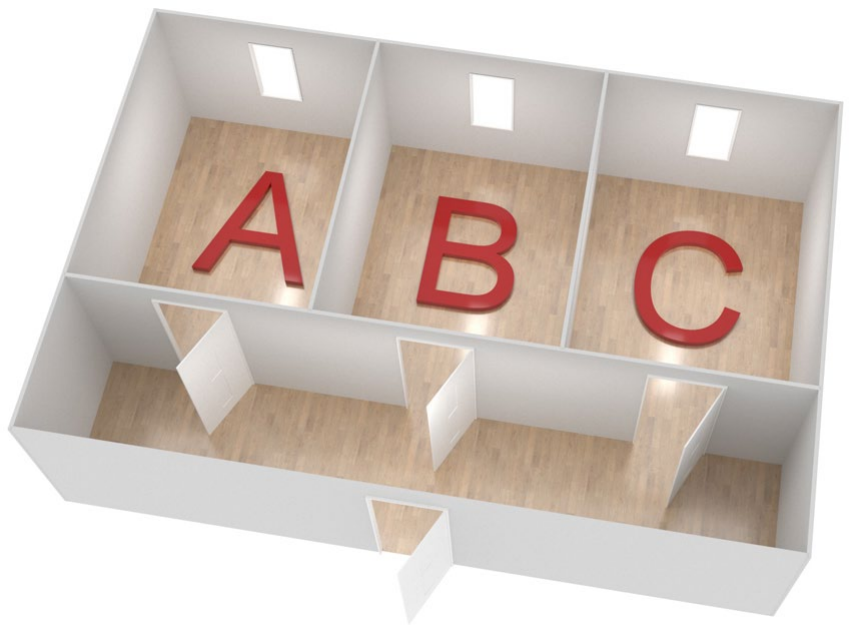
The rooms were built according to this model. Each room **(A)**, **(B)**, and **(C)** measured 3 × 4 meters.

**Figure 3:**
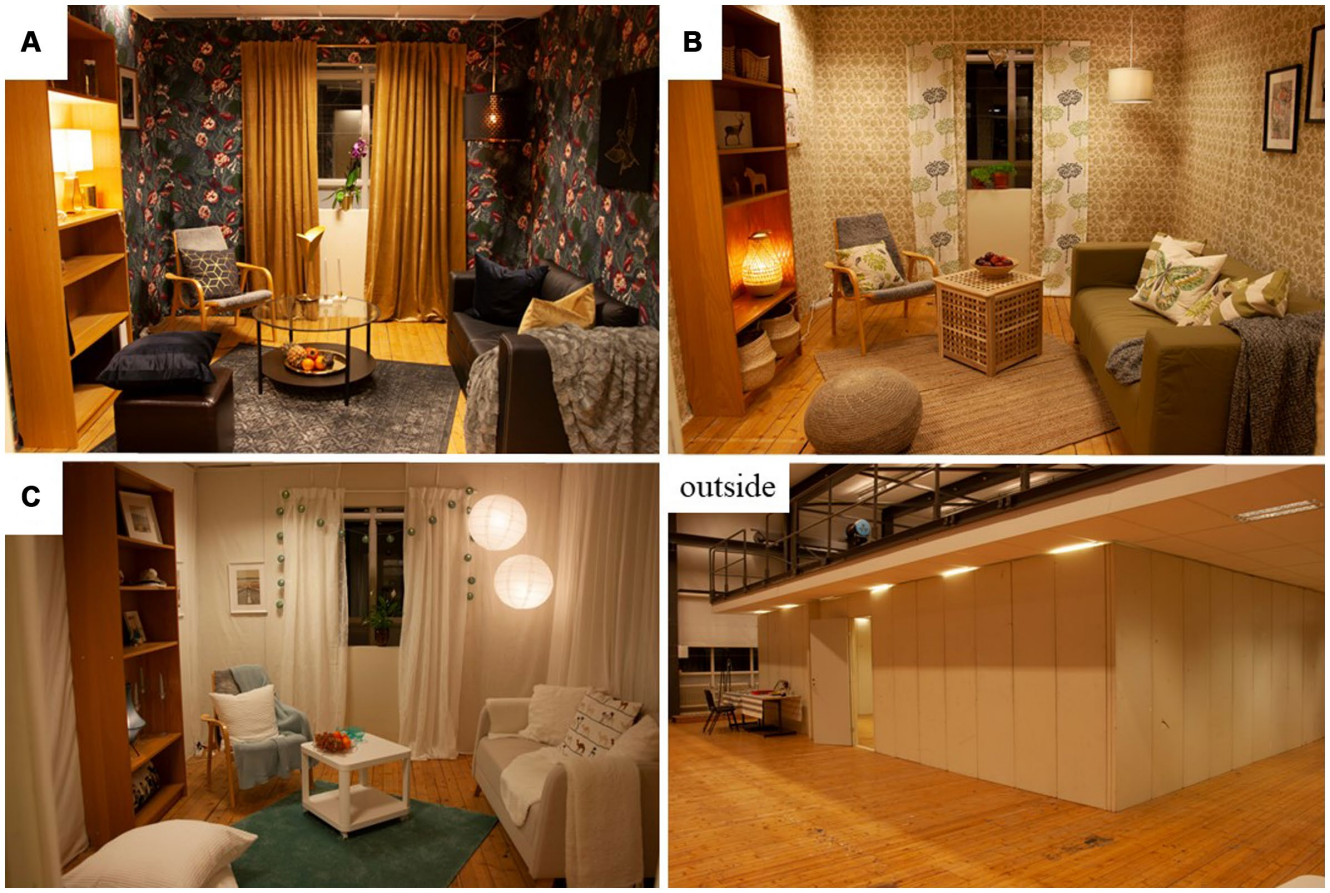
Room **(A)** the “gain” room, room **(B)** the “normative” room, and room **(C)** the “hedonic” room. The fourth picture shows the model from outside, in the full-scale lab.

Each room measured 3 × 4 meters, had a door and a window (Figure 2). The rooms were decorated to resemble “living rooms” in Scandinavian home environments (Figure 3), each furnished with a bookshelf, a chair, a sofa, a small table with a fruit bowl, curtains, carpet(s), pot-plants, ceiling lamps as well as lamps in the bookshelf, pillows or a pouf for seating, and paintings or pictures on the wall. For practical reasons, textiles were used as “wallpaper.” The rooms were decorated to prime (A) gain, (B) normative, and (C) hedonic goal frames, respectively (Figure 3). For social status the gain room was decorated in darker colors (Acking and Küller, 1969; Küller, 1975) and included business related items that can induce economic resource guarding (Kay et al., 2004) as well as golden surfaces and a gold bar piggy bank to prime for wealth rather than direct symbols for money which have been previously used in experiments (Mandel and Johnson, 2002). In the normative room, nature associations and wood surfaces were used to stimulate affection with a sense for the genuine and lasting (Küller, 1975), and in the hedonic room the aim was to achieve a cloud like interior to prime for comfort (Mandel and Johnson, 2002). For more detail on room décor, see Appendix A. The horizontal illuminance levels were measured at nine points in each room to ensure that each room had similar lighting conditions, within the range of Scandinavian home lighting.

During the main study, 83 participants provided free text responses regarding room associations. These free text responses indicated the rooms’ ecological validity. Room A (Figure 3), *the “gain” room*, was described as dark, luxurious, up-market, and expensive. It was associated with money, status, power, and riches, and non-sustainability. The room was not associated to a specific age group but was described as masculine. Room B (Figure 3), the *“normative”* room was described as the green room, associated to nature as well as to environmental consideration and sustainability. This room was mainly appraised as modern, fresh, peppy, and inspiring, but in contrast it was also described as a hypocritically “hippie” or “hipster” room. Age associations were made to young and elderly people, and the room was described as feminine. Room C (Figure 3), the *“hedonic”* room was described as a bright room, of white and/or blue. The room was largely associated with holidays, travelling, summer, the beach, “pleasure,” and freedom. The style was described as “Scandinavian,” neutral or ordinary, and uninspiring. Age associations were made to young people, and the room was described as feminine.

#### 2.3.2 The online stimulus

The online priming stimulus was an extract from an existing Instagram campaign for sustainable textile use. The Swedish Environmental Protection Agency were the senders of the campaign. At the time of the study, the campaign was no longer active. The study campaign stimulus included a collage matrix of 3 × 3 pictures from the original campaign. The selection of pictures from the campaign was based on their reach, and to capture the width of communication within the campaign. The top row pictures had received more than one thousand likes each. Two of these pictures were without text and one featured the text: “*Prolong the life of your garment – Double the amount of usage and half the climate impact*.” Another 3 pictures with more than 300 likes included the following text messages: “*Up to 6,5 kilos of chemicals are needed to create 1 kilo of clothing – Chemicals can harm people and the environment in countries where clothes are manufactured*”; “*Influence the supply! – When many ask for environmental labels the larger the chance that the shop will provide it*”; and “*Choose second hand and vintage – The clothes already exist and will not generate new emissions*.” Finally, 3 pictures without text reflected messages of the campaign: choice of material, having fun, and mending torn clothes.

Participants provided free text responses (*n* = 80) regarding their remembrance of the campaign stimulus that they had seen prior to the purchase task. Seven of these participants simply stated that they did not remember anything. Those who did recall the campaign remembered messages with a focus on sustainability and responsible consumption. The pictures were largely described as colorful and with a feeling of happiness and joy.

### 2.4 Measures

#### 2.4.1 Discrete choice experiment

To measure relative preferences towards different product attributes, an indirect elicitation method in the form of a discrete choice experiment (DCE) was utilized (Breidert et al., 2006), where participants were asked to indicate among a set of products which product they would like to purchase. The DCE mimics a real purchase situation and relies on the assumption that products can be described as bundles of attributes, and that individuals derive utility from the combination of its attributes (Train, 2009; Hensher et al., 2015). The study included two DCEs: one with t-shirts and one with bananas. The main focus is on the results from the t-shirt choices, in line with the online campaign focusing on textile use. A pilot study guided the choice of product (see Appendix A for detail). T-shirts were used in the initial DCE, since among textiles, the T-shirt is a commonly purchased and worn product. Both objects are widely available and can have sustainability related labels, and hedonic attributes (appearance) which are possible to describe in an online setting.

The t-shirt DCE was presented first in each room, followed by the banana DCE. The setup of the DCE was that participants made repeated hypothetical purchases of t-shirts, where each of the purchase situations was different and presented participants with four t-shirt options. Each t-shirt option was described by four different attributes that varied to capture each of the goal frames: a “comfortable material”-label (hedonic attribute), the price (gain attribute), “organic”- label (normative attribute), and “fairtrade”-label (normative attribute). Second, participants were presented with repeated hypothetical purchase situations of bananas, where each situation was different and included three options of different bunches of bananas. Each banana option was described by four different attributes according to the goal frames: a color perfectly yellow or with brown spots (hedonic attribute), price (gain attribute), “organic” and/or “fairtrade” labels (normative attributes). While the t-shirt DCE was undertaken prior to the banana DCE in every room, the order in which the prepared choice situations appeared for the participant was randomized to avoid ordering effects. Also, the t-shirt and banana bunch options in each situation were randomized to avoid bias associated with the option’s position on the screen.

**Figure 4:**
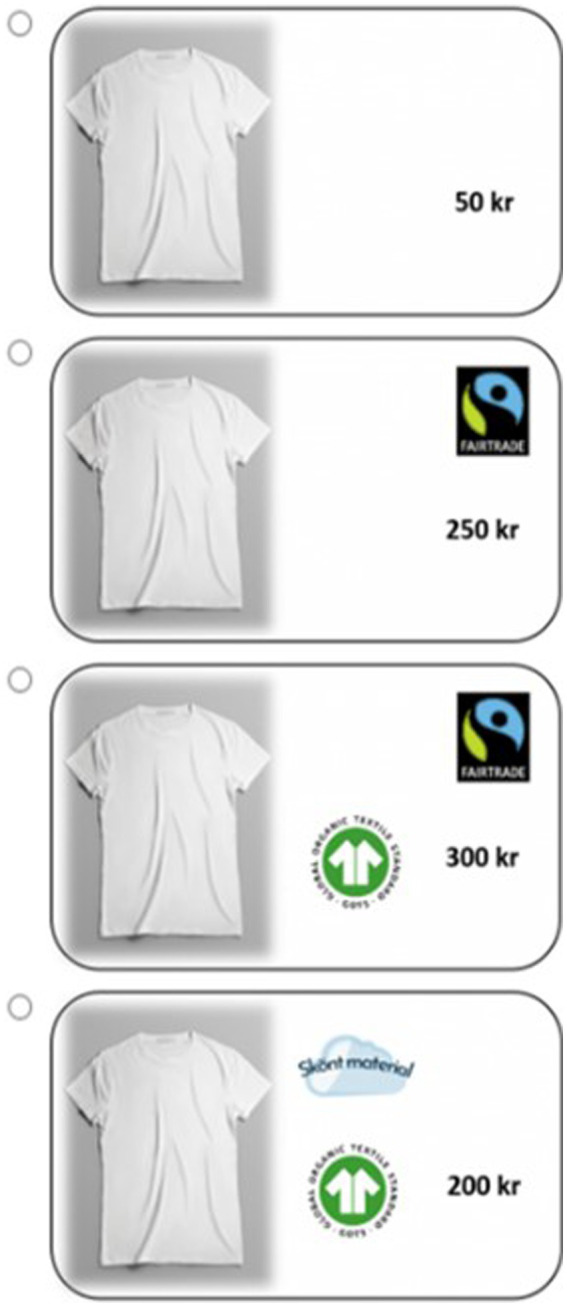
Purchase options. The picture was identical in all options, and attributes that were varied for each choice were: the “Fairtrade” label (normative), the “GOTS” label (normative), a “comfortable material” label (hedonic), and the price (gain). In each of the choice tasks there was an option to choose “If these are the only options, I refrain from purchase”.

Examples of t-shirt purchase situations are presented in Figure 4. In each situation, participants were asked to indicate which product they would like to purchase. They could only select one of the options in each situation. Participants were prompted to respond as truthfully as possible, and they had the option to not purchase anything in each of the choice situations. The hypothetical nature of the DCE implies that the tasks are not incentive compatible, which may give rise to hypothetical bias. This is a main concern when making market predictions or estimating welfare effects. However, given the focus on treatment effects from the physical environment on choice in this study, hypothetical bias is not expected to affect conclusions.

Presenting all possible combinations (full factorial design) of the four different attributes to each participant would be a demanding task. Commonly, a limited number of combinations are selected for presentation to the participants (fractional factorial design). We applied an efficiency criterion method to select which combinations of attributes to present. This method aims to find combinations of attributes that provide as small standard errors as possible, and it therefore requires prior information about the true parameters of the model. Since this is not known prior to the data collection, we applied a two-step process to generate the final experimental design (Hensher et al., 2015; Ngene Team, 2018). First, the pilot study was conducted on an initial t-shirt choice experiment design, in an online survey with a convenience sample (*n* = 37). Based on the model estimates from the pilot data, we included the parameters as priors in the generation of the final design. We applied a D-efficiency criterion for the design selection, and we used a Bayesian design to accommodate for uncertainty in the priors (Ngene Team, 2018). The final design included 24 choice situations, and these were grouped into three blocks. In each room, the participant was presented with one block of choice situations, and it was randomized which block was presented in each room. More details on the design and implementation of the choice experiment are provided in Appendix A.

#### 2.4.2 Value orientation

Participants responded to the Short Schwartz Value Survey, SSVS (Lindeman and Verkasalo, 2005), with items asking participants to rate (on a 7-point scale, ranging from “against my principles” to “important”) the extent to which ten value domains (Power, Achievement, Hedonism, Stimulation, Self-direction, Universalism, Benevolence, Tradition, Conformism & Security) act as life-guiding principles. The value domains serve as the basis for the value orientations self-transcendence and self-enhancement, which have previously been studied in relation to concerns and beliefs regarding environmental problems, and willingness to pay for eco-labels (Hansla, 2011a,b).

### 2.5 Procedure

#### 2.5.1 Lab procedures

Participants visited the lab for approximately 1 hour. Each participant was provided with an iPad, on which all survey data was collected using a web based Qualtrics (Provo, UT) questionnaire. The procedure in the lab was divided into three parts. In *the first part*, participants were seated at a desk in an otherwise unfurnished corridor outside of the three priming rooms (Figure 2) where they were provided verbal information about the study and gave their written consent to participate. They also responded to demographic questions. In *the second part* of the study, participants were subjected to the online prime and the room primes, while undertaking the choice experiment (described in detail below). All participants visited all rooms. Each participant was exposed to the online stimulus, and made purchase choices (6 choices of t-shirts and 6 choices of bananas), in each of the three rooms in one of the following orders: A-B-C (*n* = 14), A-C-B (*n* = 15), B-A-C (*n* = 15), B-C-A (*n* = 15), C-A-B (*n* = 15), or C-B-A (*n* = 14). Participants were asked to take a seat on the sofa in each room and received a brief introduction to the choice experiment. In each room, when the participants pressed the start button on the iPad, the campaign for sustainable consumption of textiles (the online stimulus) was shown on the screen for 10 s. Then, a “forward” option appeared, and the participants were asked to make their purchase choices in a simulated online shopping scenario, all within the same Qualtrics survey.

After the participant had visited, and made purchase choices, in each of the three rooms the participant was given a 15 min break but remained in the lab (outside the rooms) and were kept occupied with a puzzle. In *the third part* of the study, the participants responded to the SSVS (Lindeman and Verkasalo, 2005), rated their usual purchase choices, and provided free text responses about the online stimulus and the rooms. Participants received a cinema ticket at the value of 100 SEK for their participation.

#### 2.5.2 Analysis

To explore if preferences for different attributes depended on the room where choices were made, we estimate discrete choice multinomial logit models on the purchase data from the t-shirt and banana choice tasks, respectively. The dependent variable takes the value one for the chosen alternative in each choice task, and zero otherwise. Individuals are assumed to choose the product that provide the greatest utility for them, where the utility derived from each product depends on its attributes (Train, 2009). The preference parameters estimated in the discrete choice model are confounded with the scale of the utility, and for this reason it is only the relative size of the preference parameters that can be interpreted, not the absolute value of the parameters. However, the negative ratio of a preference parameter and the price parameter cancels out the scale term and gives the marginal willingness to pay for this attribute (Hensher et al., 2015).

To estimate the relative attribute preference in the room environments, we include interaction variables between each of the attributes and the rooms. For identification, one of the rooms is assigned as reference (the gain room), and the interaction terms indicate differences in the preference parameter depending on the room. A significance level of α = 0.05 was chosen. Models were estimated in R, with the package Apollo (Hess and Palma, 2019).

## 3 Results

### 3.1 Choice experiments

Average preference parameters for each of the t-shirt attributes are presented in the first column in Table 2. The coefficient for “organic” indicates the average preference for a product with organic label compared to a conventional product (without organic label), assuming that the products are equal in all other aspects. As shown in Table 2, individuals prefer products with a lower price over higher price (the negative price parameter). The positive preference parameters for organic and fair trade imply that individuals prefer products with such labels to non-labelled products. While the preference coefficient can be interpreted by its positive or negative sign and relative size, its exact value does not hold meaning. However, the monetary value of an attribute, the marginal willingness to pay, can be estimated from the negative ratio of the preference parameter and the price parameters, such that individuals are willing to pay an average of 77.3 SEK^1^ more for a t-shirt with the organic label over a t-shirt without the organic label. Finally, individuals prefer comfortable material-labelled t-shirts to non-labelled products.

**Table 2:** Multinomial logit model with room interactions.

			Normative room		Hedonic room	
Attribute	Coefficient	|*t*-value|	Coefficient	|*t*-value|	Coefficient	|*t*-value|
Hedonic (comfortable material)	1.41*	13.11	−0.06	0.38	0.07	0.42
Normative (organic)	1.49*	12.95	0.39*	2.33	0.25	1.51
Normative (fair trade)	1.74*	14.33	0.14	0.78	0.22	1.26
Gain (price)	−0.02*	20.37	−0.0003	0.25	−0.002	1.26
Do not purchase	−1.86*	11.39	0.29	1.30	0.07	0.29

T-shirt purchases. *N* = 88 individuals, 2,112 choices. LL = 2,130. * Indicates statistical significance at 5% level.

The main parameters of interest are the interactions between product attributes and the room where the choices were made. These are interpreted as the effects from the rooms on the choices made. The gain room was selected as the reference room in the model, and the interaction terms between each of the attributes and the normative and hedonic rooms enables us to test for room effects on preferences. The second column in Table 2 displays interaction terms for the normative room, and the third column displays interaction terms for the hedonic room compared to the gain room, indicating if preferences for product attributes were different in these rooms compared with the gain room. The statistically insignificant interaction terms of the first row implies that the hedonic attribute (Comfortable Material) is not more important (stronger preference) in the hedonic primed room than in the other rooms. The statistically insignificant interaction term for price and the hedonic/normative rooms implies that the importance of price is not different across rooms, with the gain room as reference. Notably, in line with prior expectations, we find that the positive preference parameter for organic is larger in the normative room than in the gain room. The willingness to pay for organic labelled t-shirts was 96.1 SEK in the norm-room, while it was 77.3 SEK in the gain room. No statistically significant differences for the other normative attribute (fair trade) were observed.

### 3.2 Robustness of results

To investigate the robustness of the findings in 3.1, the models in section 3.1 were re-estimated while controlling for the individuals’ value orientations. Results from the T-shirt data is presented in Table B1 (Appendix B). Controlling for value orientations, by interacting the two value dimensions with each of the product attributes, improves model fit significantly [Likelihood Ratio test statistic = 28.8 is above critical value (*p* < 0.001)]. The value orientation interaction terms are in line with theoretical expectations: organic and fair-trade attributes are relatively more important for individuals with a stronger affinity to self-transcendence than individuals with a stronger affinity to self-enhancement. Importantly, the interaction term between organic attribute and the normatively primed room remains statistically significant after controlling for the value orientations.

An additional set of analysis, which diverts from the textile focus of the information campaign in this study, but which connects with prior studies in the area, includes choice tasks with bananas. Results from the banana-choices are available in Appendix C. We note that the mean parameters are all in line with expectations; on average, individuals prefer lower prices, they prefer yellow to browned banana, and they prefer products with organic and fair-trade label over non-labelled products. Like the t-shirt experiment, the preferences for the hedonic attribute (browned bananas) and gain (price) are not different depending on the room priming. In contrast to the t-shirt choices, preferences for organic are not statistically significantly different in the normative room compared to the gain room. Results from the Banana-data, where value orientation is included, suggest that model fit improves when controlling for value orientation (LR-test *p*-value <0.05, see Table B2, Appendix B). The value orientation interaction terms suggest that fair-trade is relatively more important for individuals with a stronger affinity to self-transcendence than individuals with a stronger affinity to self-enhancement.

## 4 Discussion

This study provides insight into how cues in the in the ambient physical environment can influence consumers’ psychological states, and subsequent online purchase decisions, particularly in relation to sustainable choice behavior (Mari and Poggesi, 2013; Fischer et al., 2021). The study’s uniqueness derives from studying behavior at the interface of the physical and online environments, which has otherwise received little attention in behavioral interventions and studies of consumer behavior (Mari and Poggesi, 2013). The interdisciplinary setup that combined controlled full scale indoor environments aiming to activate different goal frames (Lindenberg and Steg, 2007), with an online choice experiment (Louviere et al., 2000) ensured a robust study design with high ecological validity. By including a sustainability campaign stimulus, the study has real-world relevance for how marketing messages could interact with environmental cues to support behavior.

The hypotheses postulated a congruence between the goal frame priming in the indoor environment and the participants’ relative preference for associated product attributes in an online purchase task. The hypotheses were only met with regards to enhanced choice of products with normative attributes in the room with a normative priming. In this room, the choice of t-shirts with organic branding was enhanced, in comparison to the participants’ choice of organic branding in the room designed to associate with money and status. This is an interesting finding – the study participants made environmentally sustainable choices to a greater extent in a physical environment congruent with the environmentally sustainable choice.

Apart from the different room environments, participants interacted with the “sustainable textile” campaign stimulus in each room prior to making their purchase choices. Assuming the campaign stimulus primed a focal normative goal frame, it is possible that the incongruent room environments (hedonic/gain) reduced the normative priming effect of the campaign, whereas the congruent room environment sustained the effect (Francken et al., 2011). Considering the potency of soft policy measures such as campaigns for encouraging behavioral change, the results illustrate the importance of matching environmental communication interventions with the contextual and situational factors of the expected receiver (e.g., Geller, 2002). In the current study, it was not possible to include a control group that did not interact with the campaign due to practical constraints and limited resources. We are therefore not able to isolate the possible effect of the campaign stimulus on purchase choice. This is a clear limitation of the current study, and future research could seek to explore further how room priming can have an effect in enhancing or masking different sustainability interventions including for instance information provision, product placement, or campaign messages.

With regards to the other normative attribute, i.e., fairtrade-labelled t-shirts seeking to promote social sustainability, no enhanced choice was observed in any room. The selection for fairtrade branding was already strong in all rooms. Furthermore, the normative room interior primarily focused on nature associations and environmental sustainability and may thus have reduced any social sustainability priming effect caused by the campaign stimulus (Francken et al., 2011). It is also possible that the priming of social sustainability was weak in both room and campaign stimuli. Since priming effects are sensitive to specificity (Srinivas, 1993; Schacter et al., 2004) any primes for environmental sustainability could fail to prime socially sustainable behavior, e.g., enhanced choice for fairtrade branding.

Priming effects are temporal states caused by associations (Custers and Aarts, 2010; Minton et al., 2017; Thøgersen and Alfinito, 2020), and are not expected to cause long-term effects on behaviors (Schacter et al., 2004). The priming effect of each room stimulus is expected to only last while the participant resides in the room, and therefore any priming caused in a specific room setting would not be expected to carry on to the situational context of the next room. To avoid unintentional bias from the room order, the presentation order of the rooms was counterbalanced. Within individuals, long-lasting and stable, almost trait-like, effects on purchase choices are likely related to individual values (Schwartz, 1994). The current study confirms the importance of individual values on purchase decisions, which is in line with prior research (e.g., Lagerkvist et al., 2023).

Furthermore, although this study does not attempt to evaluate the effect of a campaign message on purchase choices *per se*, it is noteworthy that the increased choice of environmentally sustainable (organic) products was only observed for t-shirts and not for bananas. Potentially, the discrepancy between the different products may relate to priming specificity (Srinivas, 1993; Schacter et al., 2004) and hence the sustainable textile campaign’s congruence with the t-shirt. The priming artefacts in the room itself included both textile artefacts (e.g., curtains, rugs, blankets of natural material) and fruit bowls (e.g., locally produced apples, see Figures 3A–C) and should be able to prime the association for both product categories, while the campaign was specifically targeting consumption of sustainable textile.

However, we also note that consumers are more familiar with the normative attributes of food compared to textiles, and participants may hence have pre-established behaviors with regards to the purchase of these products. As priming effects are small, the room environment may have been better able to influence choices for sustainable t-shirts if this behavior was new, but not for bananas if there was a pre-established preference for these products. We also consider that the t-shirt choice task was the primary focus of the study. In the experiment, purchase choices for bananas were made after those of t-shirts, which may have influenced the effect. Future studies could investigate if priming effects from rooms vary across product types, by including multiple products in randomized order. This will, however, require larger samples of participants.

Relatedly, it is possible that the limited sample size of this study contributes to few statistically significant parameters in the model at large, thereby leading to a type II error. Sample size in experiments of this kind is a trade-off, as data collection relies on participants visiting the lab. The methodology is labor intensive and time consuming, leading to limited recruitment within the scope of this study. However, the methodology allowed a controlled environmental representation with high ecological validity (Nasar, 2008) which was prioritized in this study. A limitation of relying on hypothetical purchases is that participants can ignore real-life consequences of their purchase choice, and this is a question that has received a lot of attention in the field of DCEs (Caputo and Scarpa, 2022). Hypothetical DCEs are associated with concerns regarding hypothetical bias, i.e., that respondents respond in a way that is different from their true preferences. Therefore, future methodological developments could include studying real-world online purchases of participants reside in controlled room environments. Such methodological development could shed light on the generalizability of results and be of importance for market predictions. However, in the current study, the main interest is to explore the treatment effects (i.e., the room effect on behavior) and therefore the hypothetical bias of DCE is of less concern, as there is no theoretical reason to expect that the bias would be different between the rooms. Future studies may also focus on a broader variety of products to increase the generalizability of results. Only including t-shirts and bananas, limits the generalizability of the current study to other product categories.

The study results may inform further steps within the field. For instance, in consumer psychology, research on environmental impact has largely focused on atmospherics and store environments regarding shopping behavior, in either physical or online settings (Mari and Poggesi, 2013). Our study suggests that additional learnings may be derived from research in environmental psychology where the theoretical and practical emphasis is to understand the effects of environmental perception and appraisal on people’s behavior (e.g., Brunswik, 1952; Maslow and Mintz, 1956; Altman, 1975; Küller, 1991). With European online shopping on the increase (Eurostat, 2022b), it is important to explore factors that may support sustainable online consumption, both in the online and the physical environment. Not the least, is this important for communicators working with the development of information and online campaigns, for whom it is essential to understand how people make meaning of the content in different situational contexts, such as the online consumer’s physical room setting (Mari and Poggesi, 2013).

## 5 Conclusion

Findings of this study suggest that the physical room environment can prime online purchase choices. However, priming effects appear specific – where the room environment triggered associations to environmental sustainability the choice of organic, but not fairtrade-labelled (social sustainability), products were enhanced. While we focus on the environmental sustainability purchase behavior, future research should investigate if the congruence between campaign stimuli and physical environment gives equally sized effects also in relation to other goal frames. For example, would gain-focused campaign stimuli give an equally large effect in the gain room, as the normative-focused campaign stimuli gave in the normative room? The current study illustrates how individual behavior can be important for the environment through environmentally sustainable choices, but also how the environment is important for sustainable human behavior (Gärling, 2014).

## Data availability statement

The raw data supporting the conclusions of this article will be made available by the authors, without undue reservation.

## Ethics statement

The studies involving humans were approved by Swedish Ethical Review Authority, Linköping. The studies were conducted in accordance with the local legislation and institutional requirements. The participants provided their written informed consent to participate in this study.

## Author contributions

AEk: Conceptualization, Data curation, Investigation, Methodology, Project administration, Supervision, Validation, Visualization, Writing – original draft, Writing – review & editing. AEd: Data curation, Formal analysis, Investigation, Methodology, Software, Writing – original draft, Writing – review & editing. JR: Conceptualization, Data curation, Funding acquisition, Investigation, Methodology, Software, Supervision, Visualization, Writing – review & editing. MJ: Conceptualization, Funding acquisition, Investigation, Methodology, Project administration, Resources, Supervision, Validation, Writing – review & editing.

## Footnotes

^1^The marginal willingness to pay is obtained from coefficients 1.4918/−0.0193.
