# Supplementary material for: The physical environment matters: room effects on online purchase decisions
Source: Front Psychol. 2024 Jun 18;15:1354419. doi: 10.3389/fpsyg.2024.1354419 (PMC11217526; doi:10.3389/fpsyg.2024.1354419)
Supplement: Supplementary file 1 [file Table_1.DOCX]

**Appendix A – Methodological Detail Appendix**

1. **Scales**

All scales were presented to participants in Swedish, below are English translations.

- 1. **The short Schwartz’s value survey**

References:

Johansson, M., Rahm, J., & Gyllin, M. (2010). Landowners' Participation in Biodiversity Conservation Examined through the Value-Belief-Norm Theory. *Landscape Research*, 38, 295-311.

Lindeman, M., & Verkasalo, M. (2005). Measuring values with the short Schwartz's value survey. *Journal of Personality Assessment*, 85, 170-178, DOI: 10.1207/ s15327752jpa8502_09

| **Please rate how important the following concepts are in our life.** | |
| --- | --- |
| *Value orientation:* | *7-point scale: Against my principles – unimportant – fairly unimportant – unsure – fairly important – important – of greatest weight* |
| *Power* | Power and authority |
| *Achievement* | Performance and success |
| *Hedonism* | Pleasure and contentment |
| *Stimulation* | Variation and excitement |
| *Self-direction* | Freedom and independence |
| *Universalism* | Tolerance and justice |
| *Benevolence* | Kindness and responsibility |
| *Tradition* | Humbleness and respect |
| *Conformity* | Conform and follow others |
| *Security* | Safety and security |

- 1. **Semantic Environmental Description, pre-test only**

References:

Küller, R. (1975). *Semantisk miljöbeskrivning (SMB)*. Psykologiförlaget, Stockholm, Sweden

Küller, R. (1979). A semantic test for use in cross-cultural studies. *Man-environment systems*, 9, 253-256.

| **Below you will find several scales where you should mark how you experience several aspects of the room that you are in.** | |
| --- | --- |
| *Dimension:* | *7-point scale: “slightly” - - - - - - - “very”* |
| *Pleasantness* | Boring (rev), brutal (rev), good, idyllic, pleasant, secure, stimulating, ugly (rev) |
| *Complexity* | Composite, lively, motley, subdued (rev) |
| *Unity* | Consistent, functional, of pure style, whole |
| *Enclosedness* | Airy (rev), closed, demarcated, open (rev) |
| *Potency* | Feminine (rev), fragile (rev), masculine, potent |
| *Social status* | Expensive, lavish, simple (rev), well-kept |
| *Affection* | Aged, modern (rev), new (rev), timeless |
| *Originality* | Curious, ordinary (rev), special, surprising |

- 1. **Perceived Restorativeness Scale (PRS), pre-test only**

Reference:

Adapted version of PRS based on Hartig et al. (1997).

Hartig, T., Korpela, K., Evans, G.W., & Gärling, T. (1997). A measure of restorative quality in environments. *Scandinavian Housing and Planning Research*, 14, 175-194.

| **How do you experience this room?** Below you will find a number of statements. Read each statement carefully and ask yourself: **To what degree is the statement in line with my own experience of this room?** | |
| --- | --- |
| There is much to discover and explore here. | *11- point scale:*  *0 (not at all) – 1 –  2 (very little) – 3 –  4 (quite little) – 5 –  6 (pretty much) – 7 –  8 (very much) – 9 –  10 (completely)* |
| Spending time here gives me a break from my everyday routines. |  |
| In this room, I get away from what usually demands my attention. |  |
| This room is fascinating. |  |
| I like this room. |  |

- 1. **Core Affect Scale, pre-test only**

References:

Västfjäll, D., & Gärling, T. 2007. Validation of a Swedish short self-report measure of core affect. *Scandinavian Journal of Psychology*, 48, 233-238.

Johansson, M., Sternudd, C., & Kärrholm, M. (2016). Perceived urban design qualities and affective experiences of walking. *Journal of Urban Design*, 21, DOI: 10.1080/13574809.2015.1133225

| **How do you feel when you are in this room, right now?** |
| --- |
| 5 – point scale:  Sad/depressed/displeased - - - - - Glad/happy/pleased  Sleepy/passive/dull - - - - - Awake/active/peppy |
| 5 – point scale (presented as a 5 x 5 grid):  Active - - - - - Passive  Negative - - - - - Positive |

- 1. **Goal Framing Association, pre-test only**

Reference**:**

The scale was developed by the authors based on Lindenberg & Steg (2007).

Lindenberg, S., & Steg, L. (2007). Normative, gain and hedonic goal frames guiding environmental behavior. *Journal of Social Issues*, 63, 117-137.

| **When I look around in this room, my associations are made to…** | |
| --- | --- |
| *Dimension:* | *7-point scale: “slightly” - - - - - - - “very”* |
| *Hedonic* | Enjoyment, undemand, pleasure, idleness |
| *Gain* | Success, money, status, riches |
| *Normative* | Solidarity, sustainability, consciousness |

1. **Choice experiment design**

References:

Caputo, V. and Scarpa, R. (2022) ‘Methodological advances in food choice experiments and modeling: current practices, challenges, and future research directions’, *Annual Review of Resource Economics*. Annual Reviews, 14, pp. 63–90.

Lagerkvist, C. J. *et al.* (2020) ‘Preferences for sustainable and responsible equity funds - A choice experiment with Swedish investors’, *J Beh Exp Finance*, 28(100406).

Ngene Team (2018) *Ngene: software tool to generate experimental designs for stated choice surveys.* Econometric Software, Inc.

The pilot study included choice tasks where respondents selected t-shirts and socks. Following the choice tasks, respondents provided information about how they perceived the choice tasks. Based on analysis of the choice data and follow-up questions we made a number of adjustments to the experiment included in the main data collection.

We proceeded with t-shirts as the textile product for the main study. A key conclusion from the follow-up questions in the pilot was that the normative labels (organic and fair trade) are not characteristics that consumers are used to see or reflect upon in their purchases of textiles. For this reason, we specified the experimental design such that in each choice task there was a t-shirt alternative at the lowest price (50SEK) and with no labels. This choice alternative then represented a feasible alternative with which consumers would be familiar. A similar approach is taken in (Lagerkvist et al., 2020). The final design included this base level alternative, as well as three alternatives that varied with respect to the other attributes. We estimated an MNL model on the t-shirt choices from the pilot study, and the estimated parameters were used as priors in the generation of the final design. We allowed for uncertainty in these priors by specifying a Bayesian efficient design (Ngene Team, 2018; Caputo & Scarpa, 2022).

We also included an additional product, where consumers are more familiar with the normative attributes (organic and fair trade), and which is a product that many consumers purchase often. The product should also be possible to purchase online, and a hedonic product attribute should be suitable to represent in an online format. Based on these criteria and with insights from previous studies on normative attributes, we identified banana as a suitable product category. We used information based on previous studies as priors when generating the experimental design for the banana choice tasks. In this choice experiment all alternatives varied freely on all attributes, and the number of alternatives presented in each choice task was three. An example of two choice tasks (one for each product) is presented below:


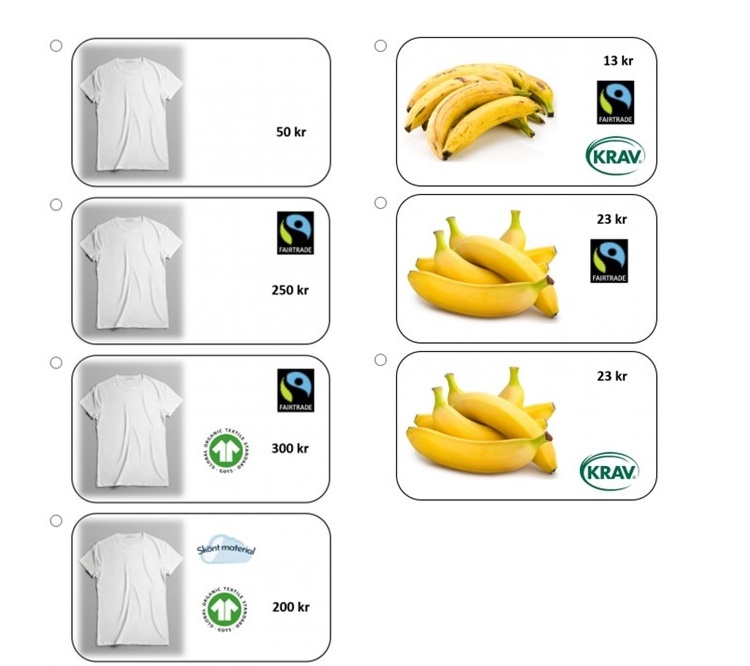


The attributes that were varied were: The “Fairtrade” label (normative), the “GOTS” label (normative), the price (gain), and the “comfortable material” label (hedonic) for t-shirts. The “Fairtrade” label (normative), an “organic” label (normative), the price (gain), and the look of the bananas – either perfect yellow or with brown spots (hedonic). In each of the choice tasks there was an option to choose “If these are the only options, I refrain from purchase”.

1. **Room stimuli**

References:

Kay, A.C., Wheeler, S.C., Bargh, J.A., & Ross, L. (2004). Material priming: The influence of mundane physical objects on situational construal and competitive behavioral choice. *Organizational Behavior and Human Decision Processes*, 95, 83-96.

Küller, R. (1975). *Semantisk miljöbeskrivning (SMB)*. Psykologiförlaget, Stockholm, Sweden

Küller, R. (1979). A semantic test for use in cross-cultural studies. *Man-environment systems*, 9, 253-256.

Mandel, N., & Johnson, E.J. (2002). When web pages influence choice: Effects of visual primes on experts and novices. *Journal of Consumer Research*, 29, 235-245.

Results from the pre-tests indicated that the “gain” room (room A in fig 2) managed to capture dimensions of Social Status in the semantic environmental description (Küller 1975; 1979). The room had darker walls, and more exclusive materials with golden surfaces, marble, and crystal glass. In this room the black sofa was made of a leather imitation, and the room had velvet cushions and curtains. To prime a “gain” goal frame, seeking to maximise one’s resources, the room also held business-related items (Kay et al. 2004) such as a leather laptop-case and document files in the bookshelf. Pictures on the walls were in gold frames or had gold patterns. The fruit bowl in the “gain room” held exotic fruit (for [Country]) available in [regional] supermarkets but clearly imported. Such fruits are often placed in a specific “exotic fruits” section in the store and demand the use of a tool for eating - a pineapple, a pomegranate, passion fruit, and khaki. In the window, an orchid in a gold-coloured pot was held as a pot-plant.

The pre-testing indicated that the “normative” room (room B in fig 2) scored slightly higher than the other rooms on the dimension of “affection” according to the semantic environmental description (Küller 1975; 1979). The room was decorated in lighter green or beige colours, textiles were green and nature patterns, furniture had wooden surfaces, and objects were to a large extent made of nature materials. Pictures on the walls were of plants and wildlife. The fruit bowl held local winter apples, a type of apple which is readily available in [Country] supermarkets in winter, but which are never imported and hence represents a local produce. In room B there was also three small recycling containers in the corner of the room. In the window, herbs in terracotta pots were kept as pot-plants.

The pre-testing revealed that the “hedonic” room, which initially combined items from show-business, travelling, and shopping, scored higher than the other rooms on “complexity” (Küller 1975; 1979). For the final design of the “hedonic” room (room C in fig 2), a decision was made to make the interior more unanimously aiming for “pleasant, soft, and easy”. In this room the interior was light with white and turquoise colours, and semi-transparent curtains were used on the walls to give an impression of softness. As the hedonic goal frame is described as “seeking pleasure and avoiding effort” the hedonic room had pictures from holidays and the seaside, and there were souvenir-like items in the bookshelf. On the light beige sofa there were white fake furs, a couple of large fluffy pillows were placed as “extra seating” on the floor, and a couple of rice-lamps lit the room to give a cloud-like impression. These items were intended to prime for “softness” similarly to the experiment by Mandel & Johnson (2002). The fruit bowl held clementines and grapes, fruits which are sweet and easy to eat, are not very exclusive or expensive, yet are clearly not local produce in [Country]. In the window, an easily managed “white sails” in a “Buddha-pot” was kept as a pot-plant.
