# Supplementary material for: The physical environment matters: room effects on online purchase decisions
Source: Front Psychol. 2024 Jun 18;15:1354419. doi: 10.3389/fpsyg.2024.1354419 (PMC11217526; doi:10.3389/fpsyg.2024.1354419)
Supplement: Supplementary file 2 [file Table_2.DOCX]

**Appendix B – analysis including value orientation**

*Table B1. Multinomial logit model with room interactions and value orientation interactions. T-shirt purchases.*

|  |  |  | Normative room | | Hedonic room | |
| --- | --- | --- | --- | --- | --- | --- |
|  | Coefficient | \|t-value\| | Coefficient | \|t-value\| | Coefficient | \|t-value\| |
| **Attribute** |  |  |  |  |  |  |
| Hedonic (CM) | 1.26* | 10.49 | - .04 | - .27 | .07 | .42 |
| Normative (Organic) | 1.72* | 12.98 | .37* | 2.19 | .27 | 1.59 |
| Normative (Fair Trade) | 1.93* | 13.92 | .10 | .55 | .23 | 1.31 |
| Gain (price) | - .02* | -19.30 | .00 | .23 | .00 | -1.33 |
| Don’t purchase | -1.86* | -11.41 | .29 | 1.28 | .06 | .26 |
| **Value: Conservation** |  |  |  |  |  |  |
| Hedonic (CM) | 0.13 | 1.76 |  |  |  |  |
| Normative (Organic) | - .21* | -2.60 |  |  |  |  |
| Normative (Fair Trade) | - .11 | -1.33 |  |  |  |  |
| Gain (price) | .00 | 1.40 |  |  |  |  |
| **Value: Self-transcendence** |  |  |  |  |  |  |
| Hedonic (CM) | - .17 | -1.94 |  |  |  |  |
| Normative (Organic) | .23* | 2.40 |  |  |  |  |
| Normative (Fair Trade) | .23* | 2.39 |  |  |  |  |
| Gain (price) | .00 | -1.43 |  |  |  |  |

N= 88 individuals, 2112 choices. LL=2116***.*** ** indicates statistical significance at 5% level.*

*Table B2. Multinomial logit model with room interactions and value orientation interactions. Banana purchases.*

|  |  |  | **Normative room** | | | **Hedonic room** | | |
| --- | --- | --- | --- | --- | --- | --- | --- | --- |
| **Attribute** | **Coefficient** | **\|t-value\|** | **Coefficient** | **\|t-value\|** | **Coefficient** | | **\|t-value\|** |  |
| Hedonic (brown) | - .82* | -5.44 | - .01 | - .04 | - .07 | | - .32 |  |
| Normative (organic) | 1.03* | 8.59 | .23 | 1.50 | .24 | | 1.55 |  |
| Normative (fair trade) | 1.06* | 8.67 | .11 | .67 | .18 | | 1.14 |  |
| Gain (price) | - .15* | -13.51 | - .02 | -1.09 | - .02 | | -1.50 |  |
| Don’t purchase | -4.00* | -13.02 | - .22 | - .49 | - .14 | | - .31 |  |
| **Value: Conservation** |  |  |  |  |  | |  |  |
| Hedonic (brown) | .15* | 2.38 |  |  |  | |  |  |
| Normative (organic) | - .13 | -1.77 |  |  |  | |  |  |
| Normative (fair trade) | - .15* | -2.08 |  |  |  | |  |  |
| Gain (price) | .01 | 1.70 |  |  |  | |  |  |
| **Value: Self-transcendence** |  |  |  |  |  | |  |  |
| Hedonic (brown) | - .07 | - .88 |  |  |  | |  |  |
| Normative (organic) | - .05 | - .64 |  |  |  | |  |  |
| Normative (fair trade) | .17* | 2.00 |  |  |  | |  |  |
| Gain (price) | - .01 | -1.77 |  |  |  | |  |  |

N= 88 individuals, 2112 choices. LL=2065***.*** ** indicates statistical significance at 5% level.*
