# Supplementary material for: The physical environment matters: room effects on online purchase decisions
Source: Front Psychol. 2024 Jun 18;15:1354419. doi: 10.3389/fpsyg.2024.1354419 (PMC11217526; doi:10.3389/fpsyg.2024.1354419)
Supplement: Supplementary file 3 [file Table_3.DOCX]

Table C2. displays results from the Banana-choices. The mean parameters for the attributes (first column) are in line with expectations. On average, individuals prefer lower prices, and they prefer yellow to browned bananas. Moreover, individuals prefer products with organic and fair-trade label over non-labelled products. Like the t-shirt experiment, the preferences for the hedonic attribute (browned bananas) and gain (price) are not different depending on the room priming. In contrast to the t-shirt choices, preferences for organic are not statistically significantly different in the normative room compared to the gain room.

*Table 2. Multinomial logit model with room interactions. Banana purchases.*

|  |  |  | Normative room | | Hedonic room | |
| --- | --- | --- | --- | --- | --- | --- |
| Attribute | Coefficient | \|t-value\| | Coefficient | \|t-value\| | Coefficient | \|t-value\| |
| Hedonic (brown) | -.71* | 5.01 | .01 | .04 | -.07 | .33 |
| Normative (organic) | 1.00* | 9.33 | .23 | 1.46 | .24 | 1.55 |
| Normative (fair trade) | .91* | 8.36 | .09 | .60 | .17 | 1.10 |
| Gain (price) | -.14* | 13.43 | -.02 | 1.08 | -.02 | 1.51 |
| Don’t purchase | -3.98* | 12.99 | -.22 | .50 | -.14 | .32 |

N= 88 individuals, 2112 choices. LL=2074***.*** ** indicates statistical significance at 5% level.*
